# Modulation of lung inflammation and immune markers in asthmatic rats treated by *Portulaca oleracea*

**Published:** 2017

**Authors:** Mahsa Kaveh, Akram Eidi, Ali Nemati, Mohammad Hossein Boskabady

**Affiliations:** 1 Department of Biology, Science and Research Branch, Islamic Azad University, Tehran, Iran; 2 Department of Biochemistry and Biophysics, Faculty of Science, Mashhad Branch, Islamic Azad University, Mashhad, I.R. Iran; 3 Neurogenic Inflammation Research Center, Mashhad University of Medical Sciences, Mashhad, Iran; 4 Department of Physiology, School of Medicine, Mashhad University of Medical Sciences, Mashhad, Iran

**Keywords:** Portulaca oleracea, Total protein, Phospholibase A2 (PLA2), Immunoglobulin E, Sensitized rat, Asthma

## Abstract

**Objective:**

Previous studies indicated anti-inflammatory effects for *Portulaca oleracea *in various inflammatory disorders. In this study, the effects of *P. oleracea* on bronchoalveolar lavage fluid (BALF) levels of total protein (TP), Phospholipase A2 (PLA2) and IgE in sensitized rats were examined.

**Materials and Methods:**

Male rats were randomly divided into six groups namely, control (group C), sensitized rats (group S), sensitized animal treated with three concentrations of the extract of *P. oleracea* and dexamethasone (n = 8 for each group). The levels of TP, PLA2 and IgE in BALF were measured.

**Results:**

The levels of TP, PLA2 and IgE were significantly increased in the BALF of group S compared to group C (p<0.001 for all cases). However, treatment of S rats with all concentrations of the extract, resulted in a significant and concentration-dependent reduction in BALF levels of TP, PLA2 and IgE compared to group S (p<0.001 for all cases except for the effect of the low extract concentration on TP). Dexamethasone treatment also led to significant reduction of TP, PLA2 and IgE (p<0.001 for all cases). The effect of dexamethasone treatment on PLA2 was significantly higher than the effects of all extract concentrations (p<0.001 for all cases). However, the effect of high extract concentration on TP and IgE was significantly higher than that of dexamethasone (p<0.001 both cases).

**Conclusion:**

The results indicated anti-inflammatory and immunomodulatory effects of *P. oleracea* in sensitized rats (as an animal model of asthma) which was equal or more marked than dexamethasone at studied concentrations.

## Introduction

*Portulaca oleracea* (*P. oleracea*) is an annual plant belonging to the Portulacaceae family which has been used in folk medicine in many countries. The plant contains flavonoids, alkaloids, coumarins, monoterpene glycosides and fatty acids as well as alpha-linolenic acid (Omega-3) (Abdel Moneim et al., 2013 ▶; Uddin et al., 2014 ▶). The use of *P. oleracea* for treatment of fever, cramps, fumigation, and burns as well as inflammatory skin rashes is documented in traditional medical books (Rasheed et al., 2004 ▶). Due to its several medicinal usages, *P. oleracea* is listed among herbal medicines in the WHO (Lim and Quah, 2007 ▶). Several pharmacological effects, including anti-inflammatory, analgesic )Chan et al., 2000 ▶; Zakaria et al., 1998 ▶; Islam et al., 1998 ▶(, wound healing )Rasheed et al., 2003 ▶(, antimicrobial )Banerjee and Mukherjee, 2003 ▶; Banerjee and Mukherjee, 2003 ▶(, antioxidant )Arruda et al., 2004 ▶; Zijuan et al., 2009(, bronchodilatory activities )Malek et al., 2004 ▶( and relaxant effect on skeletal muscle )Okwuasaba et al., 1986 ▶; Parry et al., 1987 ▶) were shown for *P. oleracea*. The effect of *P. oleracea* on Th1/Th2 balances was also demonstrated )Askari et al., 2016 ▶(. 

Airway inflammation is the main characteristic of asthma (Busse et al., 1995 ▶) and several inflammatory cells and inflammatory mediators are involved in this process (Kelly et al., 1998 ▶). Phospholipase A2 (PLA2) is released from inflammatory activated cells due to airway inflammation in asthma (Vadas and Pruzanski, 1986 ▶). An increase in PLA2 activity results in the synthesis of eicosanoids that play an important role in the inflammation process (Vadas and Pruzanski, 1986 ▶). In serum and bronchoalveolar lavage fluid of asthmatic patients, increased PLA2 activity was reported (Kashima et al., 1993 ▶). Increased serum total protein was also shown in subjects with occupational asthma. (Qureshi et al., 2009 ▶). IgE levels correlate with asthma severity and bronchial hyperresponsiveness. However, the relation between total IgE and asthma appears to be independent of allergen sensitization (Qureshi et al., 2009 ▶; Keyhanmanesh et al., 2009 ▶; Luksza & Jones, 1982 ▶).

With regard to anti-inflammatory effect of *P.oleracea*, the effects of the extract of the plant on BALF levels of PLA2, TP and IgE in sensitized rats (a rat model of asthma), were examined in the present study.

## Materials and Methods

**Animals and induction of animal model of asthma**

Male Wistar rats weighing 220 ± 50 g were kept in an animal cage with clean filtered air (Maximiser, Thorens Caging System Inc, Hazleton, PA, U.S.A) during the experiment period (Salmon et al., 1999 ▶) in the animal house of Medical school, Mashhad University of Medical Sciences, Mashhad Iran, at 22 ± 2ºC with a 12 hr light/dark cycle and water and food were available *ad libitum*. 

Rats were sensitized by OA by intraperitoneal injections of 1mg/kg ovalbumin (OA) + 100 mg Al(OH)_3_ as adjuvant in 0.9% sterile saline on days 1, 2 and 3. Animals were then exposed to 1% OA aerosol for 20 min/day with an air flow of 8 lit/min using a nebulizer (DeVilbiss PulmoSonic, DeVilbiss Health Care Ltd, Feltham, U.K) in a whole body animal exposure chamber (15 × 20 × 27 cm), with animals had normal breathing on days 6, 9, 12, 15, 18 and 21. Experiments were performed in compliance with the codes of the Institute of Laboratory Animals Resources Commission on Life Sciences and the study was approved by the Ethics Committee of Mashhad University of Medical Sciences (Code 910968).

**Studied groups**

Rats were divided into six groups in random order as follows (n=8 for each group): 

1) Control or non-sensitized animals (group C).

2) Sensitized animals (group S).

3-5) S group animals treated with 1, 2 and 4 mg/ml extract of *P. oleracea* (groups PO 1, PO 2 and PO 4).

6) S group animals treated with 1.25 μg/ml dexamethasone (group S+D). 

The extract of the plant and dexamethasone were administered in drinking water of animals during sensitization period. The animals of C and S groups were given drinking water alone. On average, each rat in all groups used 40 ml/day drinking water and there were not any significant differences in this regard among different groups.

**Preparation of plant extract**

For extract preparation, 100 g of shadow-dried *P. oleracea* leaves powder was macerated in 1000 ml ethanol 70%. The maceration was performed at laboratory temperature for 72 hr and the mixture was dried by rotary evaporator. The yield of the extract was 17.5%. The extract concentration was adjusted to 10 mg/ml by adding distilled water to the dried extract. *P. oleracea* was collected in July 2016 from Sabzevar city, Khorasan Razavi province, Iran, and a voucher sample was preserved (Herbarium No: 240-1615-12) in the herbarium of the School of Pharmacy, Mashhad University of Medical Sciences.

**Bronchoalveolar lavage fluid (BALF) preparation**

Rats were sacrificed by ketamine on day 22 of the experiment. Trachea and lungs were then dissected after opening the chest. The left lung was lavaged with 1 mL of saline five times (a total of 5 mL) through cannulated trachea. The supernatant was collected after centrifuging samples at 2500 g at 4 ºC for 10 min and immediately stored at -70 ºC until further analysis )Dong et al., 2014 ▶(.

**Measurement of BALF total protein**,** PLA2 and IgE levels**

The BALF total protein, PLA2 and IgE level were determined using the enzyme-linked immunosorbent assay (ELISA) sandwich method according to the manufacturer’s protocol using a photometric method (Abcam and Mybiosource, America).

**Statistical analysis**

Data were presented as means ± SEM. Comparisons of the results among control, sensitized and treated groups were performed using one-way analysis of variance (ANOVA) with Tukey-Kramer’s as the post-test. For statistical analysis, InStat (GraphPad Software, Inc, La Jolla, USA) package was used and a p<0.05 was considered as statistical significance.

## Results

**PLA2** **level in lung lavage**

**Figure 1:**
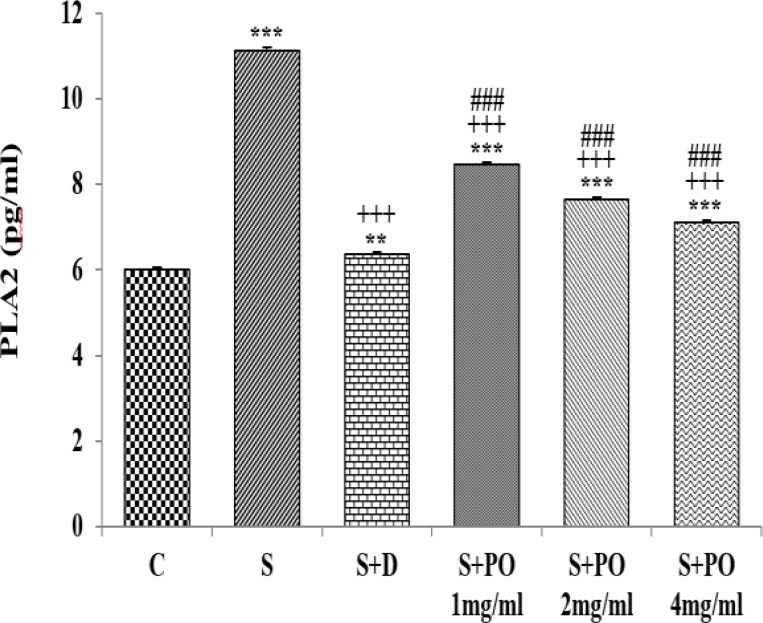
The levels PLA2 (mean ± SEM) in bronchoalveolar lavage in control (C), sensitized (S), and S animals treated with dexamethasone (S + D) and three concentrations of *P.* *oleracea* (S + PO), (n=8). Statistical significance for the difference between control and other groups:^ **^p<0.01 and ^***^p<0.001. Statistical significance for the difference between treated animals vs sensitized group: ^+++^p<0.001. Statistical significance for the difference between the data of S + PO *vs* S + D: ^###^p<0.001. The statistical comparisons were made using one-way analysis of variance (ANOVA) and Tukey–Kramer multiple post-test

BALF level of PLA2 in group S was significantly higher than group C (p<0.001, Figure 1). The PLA2 levels in all treated groups were significantly decreased compared to group S (p<0.001 for all cases, Figure 1). BALF levels of PLA2 in groups treated with all three concentrations of the extract were significantly higher than group C (p<0.01and p<0.001, respectively; Figure 1). PLA2 level in groups treated with all three concentrations of the extract were significantly lower than dexamethasone (p<0.001 for all concentrations, Figure 1). The effects of high and medium concentrations of the extract (4 and 2 mg/ml) on BALF level of PLA2 were significantly higher than the effect of its low concentration (1 mg/ml) (p<0.001 for both cases; Table 1). The effect of the high concentration of the extract (4 mg/ml) on BALF level of PLA2 was significantly higher than the effect of its medium concentration (2 mg/ml), (p<0.001; Table 1). 

**Table 1:** BALF PLA2, TP and IgE levels in groups treated with three concentrations of P. oleracea (S + PO

**Parameters**	**Control**	**S**	**S+D**	**S+PO 1**	**S+PO 2**	**S+PO 4**
**PLA2 (** **pg/ml** **)**	6.02±0.03	11.13±0.08	6.38±0.04	8.47±0.06	7.66±0.05+++	7.12±0.05 +++###
**Total protein (g/100)**	5.97±0.13	9.39±0.19	7.61±0.2	8.63±0.17	7.37±0.17 ++	5.82±0.13 +++###
**IgE**	12.77±0.22	50.56±2.22	20.54±0.85	37.14±0.74	21.24±0.51+++	11.06±0.26+++###

Values are presented as mean ± SEM. Three concentrations of *P. oleracea* were 1 (S + PO 1), 2 (S + PO 2) and 4 mg/ml (S + PO 4) (n = 8). Statistical significance for the difference between the data of S + PO 2 and S + PO 4 vs S

+ PO 1:

++ p<0.01 and

+++ p<0.001. Statistical significance for the difference between the data of S + PO 4 vs S + PO 2:

### p<0.001. The statistical comparisons were made using one-way analysis of variance (ANOVA) with Tukey–Kramer multiple post-test.

**Total protein level in lung lavage**

**Figure 2:**
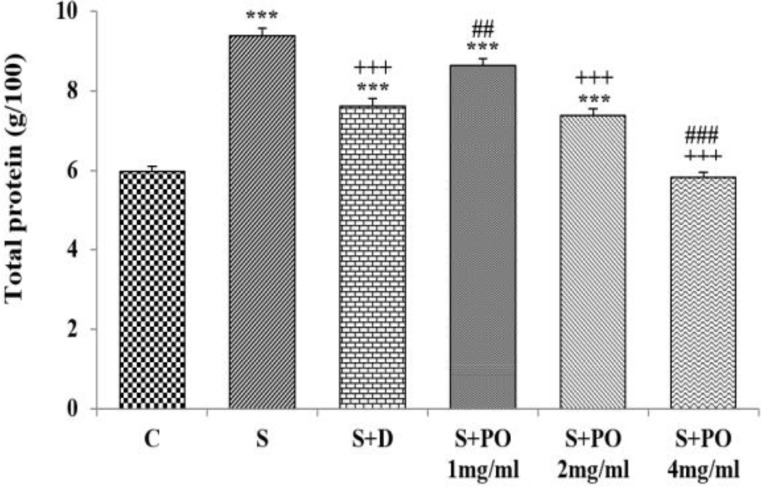
The level of total protein (mean ± SEM) in bronchoalveolar lavage of control (C), sensitized (S), and S animals treated with dexamethasone (S + D) and three concentrations of *P.* *oleracea* (S + PO), (n=8). Statistical significance for the difference between control and other groups:^ **^p<0.01 and ^***^p<0.001. Statistical significance for the difference between treated animals vs sensitized group: ^+++^p<0.001. Statistical significance for the difference between the data of S + PO *vs* S + D: ^##^p<0.01and ^###^p<0.001. The statistical comparisons were made using one-way analysis of variance (ANOVA) and Tukey–Kramer multiple post-test

BALF level of total protein in group S was significantly higher than group C (p<0.001; Figure 2). However, total protein levels in groups treated with dexamethasone and the two higher concentrations of the extract were significantly decreased compared to group S (p<0.001 for all cases, Figure 2). BALF level of total protein in groups treated with dexamethasone and the two lower concentrations of the extract were significantly higher than group C (p<0.001 for all cases; Figure 2). Total protein level in groups treated with the lowest concentration of the extract was significantly lower but the effect of its highest concentration was significantly higher than that of dexamethasone (p<0.01 and p<0.001, respectively; Figure 2). The effects of the high and medium extract concentrations (4 and 2 mg/ml) on BALF level of total protein were significantly higher than that of its lowest concentration (1 mg/ml), (p<0.001and p<0.01, respectively; Table 1). The effect of high extract concentration (4 mg/ml) on BALF level of total protein was significantly higher than that of its medium concentration (2 mg/ml), (p<0.001; Table 1). 

**IgE** **level in lung lavage**

**Figure 3:**
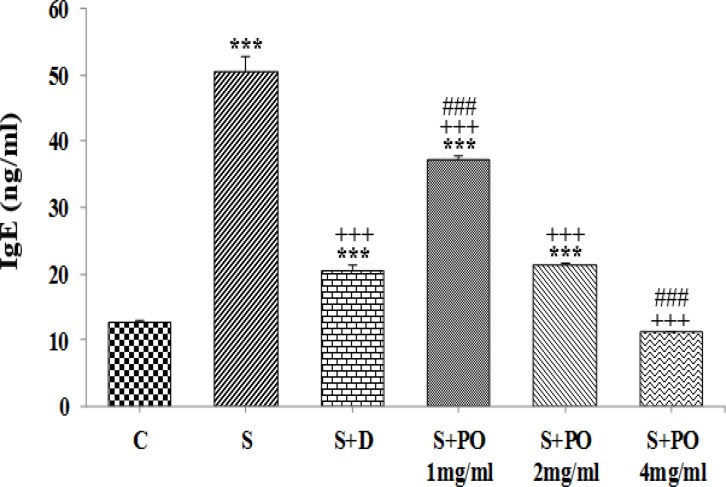
IgE level (mean ± SEM) in BALF of control (C), sensitized (S), and S animals treated with dexamethasone (S + D), three concentrations of *P.* *oleracea* (S + PO), (n=8). Statistical significance for the difference between control and other groups:^ ***^p<0.001. Statistical significance for the difference between treated animals *vs* sensitized group: ^+++^p<0.001. Statistical significance for the difference between the data of S + PO *vs* S + D: ^###^p<0.001. The statistical comparisons were made using one-way analysis of variance (ANOVA) and Tukey–Kramer multiple post-test

BALF level of IgE in group S was significantly higher than group C (p<0.001, Figure 3). The IgE levels in all treated groups were significantly decreased compared to group S (p<0.001 for all cases; Figure 3). BALF level of IgE in animals treated with dexamethasone and the two lower concentrations of the extract were significantly higher than that of group C (p<0.001; Figure 3). IgE level in group treated with the lowest concentration of the extract was significantly lower but at the highest concentration of the extract, it was significantly higher than that of dexamethasone (p<0.001 for both cases; Figure 3). The effects of the high and medium extract concentrations (4 and 2 mg/ml) on BALF level of IgE were significantly higher than the effect of its lowest concentration (1 mg/ml), (p<0.001 for both cases; Table 1). The effect of the highest extract concentration (4 mg/ml) on BALF level of IgE was significantly higher than the effect of its medium concentration (2 mg/ml), (p<0.001; Table 1). 

## Discussion

Anti-inflammatory) Chan et al., 2000 ▶; Zakaria et al., 1998 ▶; Islam et al., 1998 ▶ (and antioxidant (Arruda et al., 2004 ▶; Zijuan et al., 2009) properties of *P. oleracea* were shown previously. Therefore, in this study, the effect of the plant on lung inflammation in asthmatic rats were examined by evaluating total protein (TP), PLA2 and IgE levels in the lung lavage of sensitized rats (an animal model of asthma) treated with the extract of *P. oleracea* and compared to untreated asthmatic animals. 

The results showed increased levels of total protein, PLA2 and IgE in BALF of sensitized rats compared to control animal which indicate animal sensitization. Indeed, the results of the present study were similar to previous studies using similar method of sensitization (Keyhanmanesh et al., 2010 ▶; Neamati et al., 2009 ▶). In addition, several lung and systemic changes, similar to those seen in asthmatic patients, were reported in sensitized rats using similar method of sensitization (Shakeri et al., 2017 ▶; Shakeri & Boskabady, 2017 ▶).

In fact, increased levels of TP in BALF was also shown in subjects with occupational asthma (Qureshi et al., 2009 ▶), which may be due to increased γ globulin, C reactive protein and other inflammatory mediators with a protein structure. Increased serum IgE level in both asthmatic patients (Ahmad Al Obaidi et al., 2008 ▶) and sensitized animals (Boskabady et al., 2014 ▶) were also shown. All these studies support the findings of the present study and confirm the induction of animal model of asthma (animal sensitization).

BALF levels of PLA2 and IgE were decreased in asthmatic rats treated with all concentrations of the extract in a concentration-dependent manner. The level of TP in the BALF was also decreased due to treatment with the two higher concentrations of the extract.

The effects of the extract of *P. oleracea* on reduction of BALF PLA2 and TP levels in sensitized rats suggest the preventing effect of the plant on lung inflammation in sensitized rats. Reduction in IgE level in the BALF due to *P. oleracea* treatment also suggest an immunomodulatory effect for the plant in asthma. Since previous studies indicated that *P. oleracea* can improve serum TNF-α and IL-6 concentrations and enhance the level of LPL mRNA in liver tissue, perhaps *P. oleracea* can prevent cardiovascular diseases and atherosclerosis by these mechanisms (Xio et al., 2004 ▶). In addition, antinociceptive, anti-inflammatory (Rao et al., 2012 ▶) and immunomodulatory effects of *P. oleracea *on isolated human lymphocytes (Askari et al., 2016 ▶) have been previously demonstrated previously which support the results of the present study. 

Treatment of asthmatic rats with dexamethasone also resulted in a significant reduction in PLA2, TP and IgE which also support the anti-inflammatory and immunomodulatory effects of the studied plant. Dexamethasone, a known anti-inflammatory drug, was used in the present study as a positive control. The inhibitory effect of dexamethasone on airway inflammation in asthmatic mice have been shown (Tang et al., 2011 ▶). However, the effects of the highest concentration of the extract on PLA2, TP and IgE levels were significantly higher than those of dexamethasone. These results showed comparable or even more marked imunomodulatory effects of the extract of *P. oleracea* on lung inflammation in a rat model of asthma compared a known anti-inflammatory drug, dexamethasone. The dose of dexamethasone (1.25 μg/mL in drinking water of animals) used in this study, was 0.25 mg/kg/day during a 21-day sensitization period which was equal or even higher than previous studies. In previous studies, dexamethasone 0.5 mg/kg/day was given orally for only 3 days during sensitization period (Salama et al., 2012 ▶; Dong et al., 2014 ▶) or 1mg/kg/day was given intraperitoneally for 7 days in a 77-day sensitization period to mice (Kianmehr et al., 2017 ▶).

The effects of the extract on BALF levels of PLA2, TP and IgE were concentration-dependent. The effects of the high and medium concentrations of the extract on BALF levels of TP, PLA2, and IgE were significantly higher than its lowest concentration. The effects of the highest concentrations of the extract on BALF levels of the above-mentioned variables were significantly higher than its medium concentration. This concentration-dependent effect of the extract also supports its anti-inflammatory and immunomodulatory potential in a rat model of asthma. 

Tracheal smooth muscle relaxant effect (Boskabady et al., 2004 ▶; Oluwol and Oyediji, 2007 ▶; Parry et al., 1988 ▶) and antitussive property (Boroushaki et al., 2010 ▶) were also demonstrated for the extract of *P. oleracea* with various possible mechanism(s) including stimulatory effect on β-adrenoceptors (Hashemzehi et al., 2016 ▶) or anticholinergic property (Boskabady et al., 2016 ▶). Therefore, *P. oleracea* could combat asthma by both bronchodilatory effects, and preventive (reducing airway inflammation and immunomodulation) mechanisms. Anti-inflammatory and bronchodilatory effects of *P. oleracea*, in asthmatic patients (Chan et al., 2000 ▶; Malek et al., 2004 ▶) have been shown previously which support these findings.

In conclusion, this study showed anti-inflammatory and immunomodulatory effects of the extract of *P. oleracea* on asthma by reducing BALF levels of PLA2, TP and IgE in a rat model of asthma which suggest a potential preventive therapeutic potential for the extract of *P. oleracea* on asthma.
